# Comparing the responses of the UK, Sweden and Denmark to COVID-19 using counterfactual modelling

**DOI:** 10.1038/s41598-021-95699-9

**Published:** 2021-08-11

**Authors:** Swapnil Mishra, James A. Scott, Daniel J. Laydon, Seth Flaxman, Axel Gandy, Thomas A. Mellan, H. Juliette T. Unwin, Michaela Vollmer, Helen Coupland, Oliver Ratmann, Melodie Monod, Harrison H. Zhu, Anne Cori, Katy A. M. Gaythorpe, Lilith K. Whittles, Charles Whittaker, Christl A. Donnelly, Neil M. Ferguson, Samir Bhatt

**Affiliations:** 1 MRC Centre for Global Infectious Disease Analysis, Imperial College London, London, UK; 2 Jameel Institute for Disease and Emergency Analytics, Imperial College London, London, UK; 3 Department of Mathematics, Imperial College London, London, UK; 4 Department of Statistics, University of Oxford, Oxford, UK

**Keywords:** Computational models, Epidemiology

## Abstract

The UK and Sweden have among the worst per-capita COVID-19 mortality in Europe. Sweden stands out for its greater reliance on voluntary, rather than mandatory, control measures. We explore how the timing and effectiveness of control measures in the UK, Sweden and Denmark shaped COVID-19 mortality in each country, using a counterfactual assessment: what would the impact have been, had each country adopted the others’ policies? Using a Bayesian semi-mechanistic model without prior assumptions on the mechanism or effectiveness of interventions, we estimate the time-varying reproduction number for the UK, Sweden and Denmark from daily mortality data. We use two approaches to evaluate counterfactuals which transpose the transmission profile from one country onto another, in each country’s first wave from 13th March (when stringent interventions began) until 1st July 2020. UK mortality would have approximately doubled had Swedish policy been adopted, while Swedish mortality would have more than halved had Sweden adopted UK or Danish strategies. Danish policies were most effective, although differences between the UK and Denmark were significant for one counterfactual approach only. Our analysis shows that small changes in the timing or effectiveness of interventions have disproportionately large effects on total mortality within a rapidly growing epidemic.

## Introduction

**Figure 1:**
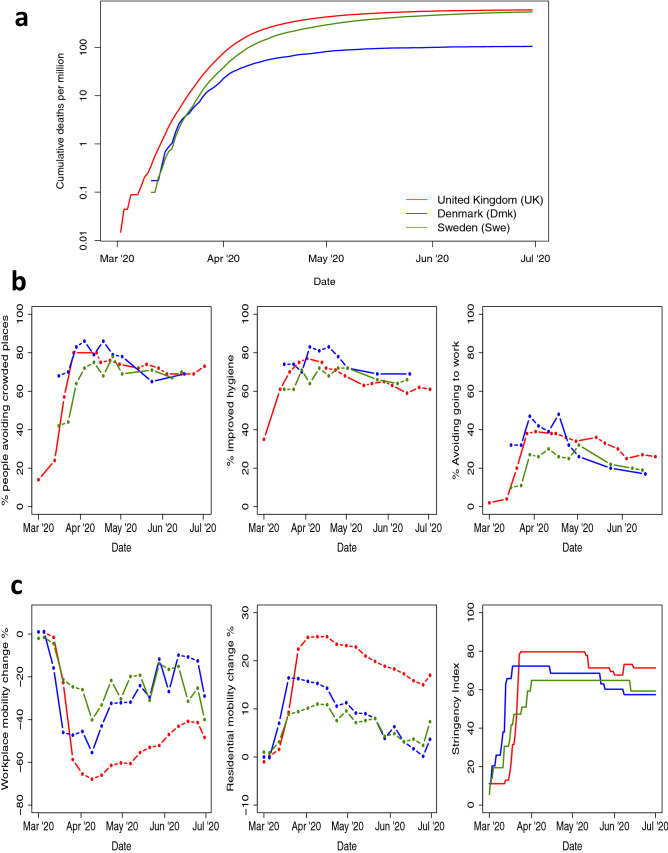
Responses to the COVID-19 epidemic in the UK (red), Denmark (Blue) adn Sweden (Green). (**a**) Cumulative laboratory-confirmed COVID-19 deaths per million by date of death. (**b**) Results of YouGov surveys of population behavioural responses to COVID-19^1^. The percentage of people: avoiding crowded public places (left); who report improved personal hygiene, e.g. frequent hand washing/using hand sanitiser (centre); avoiding going to work (right). (**c**) Google COVID-19 community mobility report data^2^ giving change from baseline in mobility associated with workplaces (left), and in residential mobility (i.e. time spent at home) (centre). Right plots the OxCGRT COVID-19 intervention stringency index^3^ measuring the number of COVID-19 policies present at any given time.

The different policy responses to COVID-19 between Sweden and other European countries have generated much discussion. Sweden relied heavily on voluntary recommendations, while most other countries favoured mandatory large-scale social distancing “lockdown”). Comparing the cumulative per-capita COVID-19 mortality in Sweden to that of Denmark and the UK is informative (Fig. 1a). While suppression of the epidemic (a reduction in the time-varying reproduction number $R_t$ to below 1) was achieved in all three countries, mortality varied substantially. Initially, Denmark and Sweden had similar epidemic trajectories, suggesting similar levels of infection seeding in each country. Following the introduction of controls between March 13th and 18th, Denmark’s epidemic flattened more rapidly than Sweden’s, and by August Sweden had a five-fold higher mortality than Denmark. The UK trajectory differed from those Scandinavian countries in its higher early mortality, suggesting earlier or greater infection seeding. Nevertheless, despite its higher infection prevalence prior to the introduction of controls, by August the UK’s cumulative mortality was similar to Sweden’s.

Behavioural data (Fig. 1b,c) suggest that the major difference between Sweden, the UK, and Denmark was the rapidity with which population contact rates were reduced, rather than the extent of this reduction. Self-reported behaviour^1^, data on population mobility^2^, and tracking of governmental interventions^3^ all tell a consistent story (Fig. 1b,c): in March, during the critical early period of exponential growth, changes in behaviour and policy in Sweden were less dramatic than those seen in the other countries shown. However, by April, the behaviour of the Swedish population had caught up, and by June stringency measures of government policy were more similar to the UK or Denmark. Although lockdown in the UK led to greater changes in mobility and higher intervention stringency than in Denmark and Sweden, these changes happened a critical few days later than in Denmark (Fig. 1c).

Here we build on this qualitative comparison of mortality trends, and use counterfactual analysis to explore how the final death toll is influenced by both the effectiveness of interventions, and the timing of interventions relative to the stage of the epidemic in each country. We choose to focus on three countries: the UK, Denmark and Sweden. Denmark and Sweden are neighbours with demographic, social and economic similarities, and both countries experienced similar initial profiles of per-capita COVID-19 mortality (Fig. 1a). However, policy responses to COVID-19 differed markedly between the two countries, and from late March COVID-19 mortality trends diverged. We include the UK in our analysis because it is another Northern European country that experienced similar cumulative per-capita COVID-19 mortality to Sweden, while adopting control policies similar to those of Denmark.

We address the counterfactual question: how would the Swedish approach to COVID-19 management have affected the epidemics of Denmark and the UK? Conversely, what impact would the policies of those two countries have had on the Swedish epidemic? Our aim is to inform future decision-making by illuminating how the timing and effectiveness of interventions interacted to influence the final disease burden experienced by a country. Since countries are unique, and randomised trials of such population-wide policies are impossible, tackling this question requires counterfactual modelling. Here we use a simple data-driven approach, which makes no prior assumptions about the effectiveness of individual policies, and which preserves apparent differences between countries in the trajectories of their early epidemics, prior to the introduction of social distancing measures. We consider only the first wave of transmission (up to July 2020), encompassing the initial adoption of policies to achieve suppression of transmission and the early relaxation of those policies. We use a Bayesian statistical approach to propagate uncertainty and an established semi-mechanistic transmission model^4^, and consider two approaches to evaluating each counterfactual scenario. While we cannot fully encompass the myriad of differences between each country, our analysis is nonetheless informative on best practice for control of future waves of the COVID-19 pandemic.

## Methods

The challenge in proposing a plausible counterfactual model is preserving the specific properties of each country, such as the basic reproduction number $R_0$ and the initial seeding of infection into the country, while transposing the impacts of policy-driven population behaviour change on transmission. For example, a plausible counterfactual scenario for the UK must preserve the fact that London is an international transport hub, which therefore experienced a high level of seeding of new infections from flights at the beginning of March^5^.

We start by fitting a semi-mechanistic model of disease transmission to the first wave of the epidemic, in all three countries spanning the period February to July 2020. The primary quantity we estimate is the time-varying reproduction number, $R_t$, using daily death data. In addition, we estimate an initial level of external seeding of infection into each country. We use a random walk to capture the changes in disease transmission due to non-pharmaceutical interventions (NPIs). It is important to note that these differences in $R_t$ encompass changes in population behaviour, and external drivers such as infection seeding from flights, and not only governmental behavioural edicts. In all models we assume that $R_t$ is fixed at $R_0$ until 13th March 2020. This assumption is necessary to prevent artefacts in $R_t$ that could arise from poor death reporting and lack of testing at that time^6^. With these estimates, we consider two approaches to projecting counterfactuals.

### Absolute $R_t$ approach

Starting on 13th March 2020, the week in which substantial social distancing measures were introduced in most European countries, and continuing until June 2020, we replace the values of $R_t$ in the ‘recipient’ country (e.g. UK) with those of the ‘donor’ country (e.g. Sweden) on the same day. The values of $R_0$ and infection seeding originally estimated for the recipient country remain unchanged. We then simulate the epidemic in the recipient country with the modified $R_t$ profile. This counterfactual represents the true course of the epidemic until 13th March, after which the recipient experiences the transmission intensity of the donor.

### Relative $R_t$ approach

Our second approach is nearly identical to the first, except that instead of swapping exact values of $R_t$, we consider relative reductions in $R_t$ starting on 13th March 2020. Hence we apply the observed donor country’s relative reductions in $R_0$ (i.e. $\frac{R_t}{R_0}$) after that date to the recipient country’s $R_0$ by simple multiplication. This counterfactual represents the true course of the epidemic until 13th March, after which the recipient experiences the relative reductions in transmission intensity of the donor.

We apply both approaches to examine the effect of Sweden, Denmark and the UK each adopting each other’s policies. The notation we use to describe counterfactuals is **donor** $\rightarrow$ **recipient**. Specifically the counterfactuals estimated are **Sweden** $\rightarrow$ **UK**, **Sweden** $\rightarrow$ **Denmark**, **UK** $\rightarrow$ **Sweden**, **UK** $\rightarrow$ **Denmark**, **Denmark** $\rightarrow$ **UK** and **Denmark** $\rightarrow$ **Sweden**. Full model details and assumptions are given in Supplementary Information Section S2.

## Results

**Figure 2:**
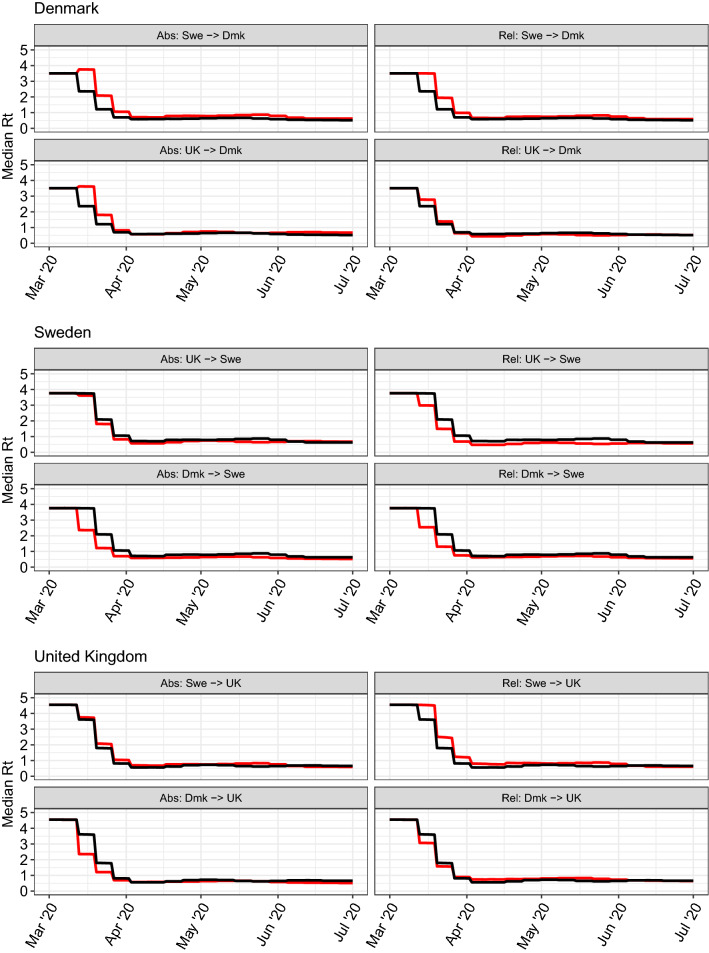
Median time-varying reproduction number over time ($R_t$) for Denmark (top block), Sweden (middle block) and the UK (lower block). Counterfactual $R_t$ profiles are in red and original fitted $R_t$ profiles are in black. For clarity, credible intervals are not shown, (see Figure S3 for uncertainty). Left column shows results for the absolute $R_t$ transposition approach, right column for the relative $R_t/R_0$ transposition approach.

**Figure 3:**
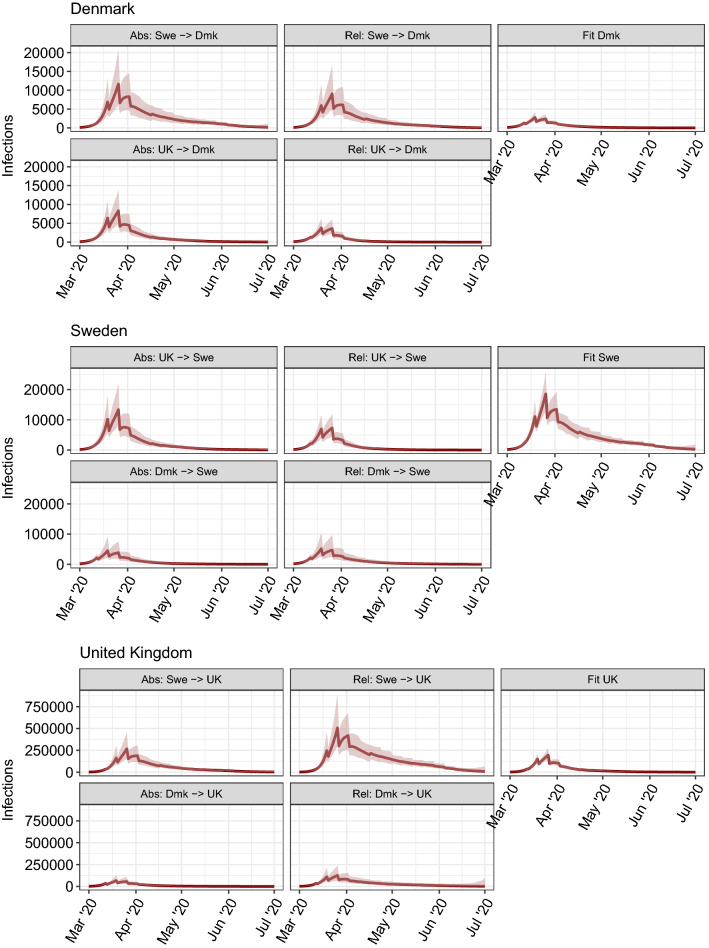
Median daily infection incidence with $95\%$ credible interval for Denmark (top block), Sweden (middle block) and the UK (lower block). Left column shows results for the absolute $R_t$ transposition approach, middle column for the relative $R_t/R_0$ transposition approach, and right column shows the fit to observed data (i.e. without counterfactual swapping). Plots show y-axis on the same scale to highlight differences between scenarios.

**Figure 4:**
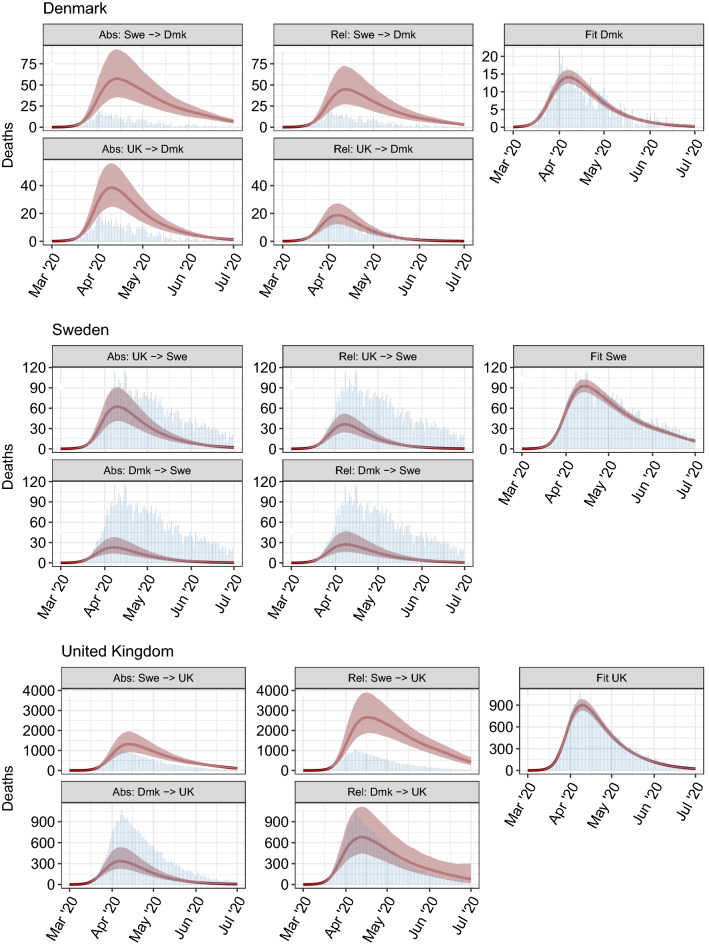
As per Fig. 3 but for daily deaths (red curves with shaded 95% credible intervals). Observed deaths are shown as blue bars.

Figure 2 shows, for each **donor** $\rightarrow$ **recipient** scenario, counterfactual $R_t$ profiles together with the original fitted $R_t$ profiles for each country. Figure 3 shows the counterfactual daily infection incidence profiles, together with the original estimates for each country. These plots demonstrate both the rapidity with which infections rise prior to the introduction of controls, but also how relatively small differences in $R_t$ can lead to large differences in the number of infections. Figure 4 shows counterfactual daily deaths for each country.

The UK experienced the highest per-capita mortality (Table 1), followed closely by Sweden, with Denmark considerably lower. The UK also experienced the highest total number of deaths (Fig. 4, Table S1). We estimate that if Denmark had adopted Swedish policies, and introduced them at the same stage of its epidemic, mortality would have been between three and four (Table 1) times higher, and thus Denmark would have experienced similar per-capita mortality to Sweden. If Denmark followed UK policies, our relative approach estimates that mortality would not have been markedly different, although our absolute approach implies that mortality would have been more than twice that observed (1). If Sweden had adopted Danish policies, both the absolute and relative approaches imply that there would have been approximately one fifth as many deaths. Sweden adopting UK policies would have resulted in a two- to four- fold reduction in mortality (Table 1). Had the UK adopted Swedish policies, deaths would have increased deaths by a factor of between 1.6 and 4 (Table 1). The UK adopting Danish policies would have reduced deaths by three fold with the absolute approach, but would have made little difference according to the relative approach (Table 1), although there is considerable uncertainty in the latter.

**Table 1:**
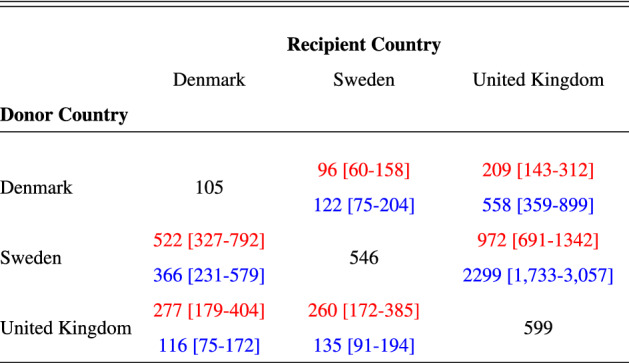
COVID-19-attributed deaths per million for the UK, Denmark and Sweden until 1st July 2020. Diagonal elements show observed mortality (black text), off-diagonal elements show mortality (median with 95% credible intervals) for counterfactual scenarios. Top red text in each row denotes counterfactuals estimated using the absolute $R_t$ transposition approach. Bottom blue text in each row denotes counterfactuals estimated using the relative $R_t/R_0$ transposition approach.

Two predominant factors explain the differences between the observed data and the counterfactual scenarios: the timing of interventions and their effectiveness, i.e. the extent to which these interventions reduced transmission. Rapid epidemic growth in all three countries in March ensured that small changes in either factor can have disproportionately large effects on total mortality.

Looking first at the absolute $R_t$ transposition approach, Denmark introduced major social distancing policies a few days earlier than both of the other two countries^4,7^. However, with an epidemic doubling time of 3–4 days^8^, a 3-day difference in the introduction of measures can lead to twofold differences in mortality. Second, in Sweden, $R_t$ took slightly longer to fall below 1 than in the UK, so when this profile is applied to the UK, its high prevalence on March 13th generated substantially more secondary infections than were seen after the policy was actually adopted. The converse is true for Sweden: the UK’s reduction of $R_t$ was slightly faster than Sweden’s, and even this small difference translates into large reductions in predicted mortality. Had the UK adopted Danish policies, mortality would have been more than halved, owing to the earlier declines in $R_t$ seen in Denmark. If Denmark had adopted Swedish policies, we estimate that its per-capita mortality would have seen comparable to Sweden’s.

Our second approach to estimating counterfactuals—swapping relative changes in $R_t$ after 16 March—gives qualitatively similar results for the Sweden–Denmark and UK–Sweden comparisons, although there are some discrepancies for the Denmark–UK comparison. The discrepancies arise from the differences in $R_0$ estimates between the three countries we consider: UK being highest, Sweden next and Denmark the lowest. Thus applying the Swedish relative changes in $R_t$ to the UK gives higher resulting $R_t$ values after 13 March, leading to even greater ongoing transmission and mortality. The converse is true applying UK policies to Sweden. Differences between the impact of policies in Denmark and the UK are much smaller with the relative approach than the absolute one, with UK policies estimated to have been marginally slower than Danish ones in reducing $R_t$ in March, but to have achieved a slightly lower $R_t$ value by April.

## Discussion

Denmark, Sweden and the UK all managed to suppress the first wave of their respective epidemics. However, the timing and effectiveness of interventions varied substantially between those countries. Our counterfactual profiles show a remarkable contrast between the small differences in the time taken to suppress $R_t$ to below 1 [Fig. 2] and the large differences in resulting death tolls [Fig. 4, Table 1]. The counterfactuals therefore demonstrate that small changes in the timing or effectiveness of intervention policies can lead to large changes in the resulting cumulative death toll, especially in the context of the rapid exponential growth initially seen in the UK, Sweden and Denmark. This result reinforces earlier work demonstrating that minimising COVID-19 mortality in the face of exponentially growing case numbers requires early and effective interventions^9–11^.

Measures adopted in Sweden relied upon voluntary population adherence with government recommendations. We implicitly assume in this analysis that the UK and Danish populations would have been as adherent to non-mandatory recommendations as was Sweden’s. Given that population trust in public institutions is higher in Scandinavia than the UK^12^, this may be an optimistic assumption for the UK.

Our analysis indicates that while all three countries successfully suppressed COVID-19 transmission, the slightly lower effectiveness of Sweden’s policies yielded substantial differences in predicted final mortality. There was more limited evidence of a smaller difference in intervention effectiveness between the UK and Denmark. While Denmark may have had marginally more effective and timely policies, the main reason that the UK saw substantially higher per-capita mortality was that infection prevalence was higher when interventions were introduced. This may have been caused by a higher $R_0$ in the UK, and/or higher levels of infection seeding into the UK.

The differences between estimates from our absolute and relative approaches can be explained by the different $R_0$ estimates in each country. Mechanistically, most social distancing interventions have a multiplicative effect on $R_0$, suggesting our relative approach is the most plausible. However, because estimates of $R_0$ and initial infection seeding are highly co-linear, it may be that the differences in our estimated $R_0$ values at least partly reflect differences in infection seeding. Nevertheless, demographic and cultural differences between the UK and Sweden (e.g. in the proportion of one-person households^13^ $\sim$50% in Sweden compared to $\sim$ 30% in the UK) give some mechanistic underpinning to genuine differences in $R_0$.

While heterogeneities in population density, age distribution and infection seeding are captured in the $R_0$ values of each country (which are unchanged in each counterfactual scenario), both the relative and absolute approaches implicitly assume that the populations of each country would respond in the same way to each set of interventions. This is unlikely to be completely valid, for example due to differences in the above factors, in household size distribution or in the willingness to adopt social distancing measures. Therefore our counterfactual scenarios should be interpreted as a exchange of both population behaviour and government policy between donor and recipient countries. Our analysis does not depend on the relative contributions of population behaviour or government action to a given policy’s success or failure.

Our analysis makes no assumptions about the mechanism of interventions, the relative contribution of individual measures, or the extent to which reductions in transmission were driven by spontaneous behaviour change rather than mandatory rules. Results are also robust to different infection fatality ratio estimates (Supplementary Material section S6). There are a number of technical limitations. First, as noted above estimates of the basic reproduction number $R_0$ are intrinsically confounded with estimates of infection seeding in each country, leading to uncertainty in $R_0$ estimates. Second, we estimate changes in transmission rates, $R_t$, from the incidence of deaths. While our analysis uses a large dataset (Fig. S1) to estimate the onset to death delay distribution, the high variance of this distribution leads to high uncertainty in $R_t$ estimates (Fig. S3), even when only estimating weekly mean $R_t$ values. This uncertainty could theoretically be reduced using laboratory-confirmed case incidence data. However, variation in testing criteria and capacity between countries and over time make trends in case incidence harder to interpret reliably than trends in deaths. Third, national-level $R_t$ estimates average over a high level of geographic, social and demographic heterogeneity in transmission within each country. It is unclear how such heterogeneities between countries would determine the relative success of a country’s COVID-19 response, as opposed to differences in policy. This can be circumvented to some extent by interpreting each counterfactual scenario as an exchange of population behaviour as well as policy, although it is still true that not every country would respond in exactly the same way to each intervention.

Our aim has been to compare the responses of different countries to their first wave of the COVID-19 pandemic, so that we might learn how best to control future waves, or indeed future pandemics. It is important to stress that we have had the benefit of hindsight, and considerably more is known about the virus than was known at the beginning of the pandemic. Decision-makers in every country had the near-impossible task of choosing to enact or refrain from a range of policy options, each of which could benefit or harm different sections of their societies in different ways, during a rapidly unfolding global pandemic. In addition, uncertainty in the lethality, morbidity and transmissibility of SARS-CoV-2 was considerably greater than is now the case.

Our key conclusion is that under conditions of high growth and high infection prevalence, small differences in the timing and effectiveness of control strategies have dramatic effects on the resulting numbers of cases and deaths. Implementation of prompt and effective interventions minimised mortality. Denmark saw low mortality because it reacted early with highly effective interventions at a time of relatively low infection prevalence. The much higher mortality seen in the UK and Sweden occurred for different reasons. In Sweden, despite conditions that were initially more favourable, higher mortality resulted from interventions bringing $R_t$ to below 1 less quickly than was achieved elsewhere. The UK suffered because its epidemic had progressed further than in many countries by the time effective interventions were implemented. For the same reason it is also true that even if the UK had intervened at the same time as Denmark, its mortality would have exceeded Denmark’s. These conclusions hold lessons for management of current increases in transmission being seen across Europe.

## Supplementary Information

Supplementary Information.

## Data Availability

All models are fitted using the R package Epidemia available at https://imperialcollegelondon.github.io/epidemia^14^. Full source code and raw data used in this study can be found online at GitHub link https://github.com/ImperialCollegeLondon/covid19model/tree/whatif/whatif. Official counts of laboratory-confirmed deaths by date of death for the UK was obtained from the UK government dashboard^15^, for Sweden via the Public Health Agency of Sweden (Folkhälsomyndigheten)^16^ and for Denmark from the State Serum Institute (Statens Serum Institut)^17^.
