# Supplementary material for: Comparing the responses of the UK, Sweden and Denmark to COVID-19 using counterfactual modelling
Source: Sci Rep. 2021 Aug 11;11:16342. doi: 10.1038/s41598-021-95699-9 (PMC8358009; doi:10.1038/s41598-021-95699-9)
Supplement: Supplementary file 1 — Supplementary Information. [file 41598_2021_95699_MOESM1_ESM.pdf]

Supplementary Material: Comparing the responses of the UK, Sweden and Denmark to COVID-19 using counterfactual modelling

S1 Onset-to-Death distribution

Key to estimating R_t is the onset-to-death distribution. The results of[1] are updated in this paper with new data and improved methods. We use data from the COVID-19 Hospitalisation in England Surveillance System (CHESS) up to 25th September 2020. We filter data as follows

- date of onset of symptoms $\geq 2020/03/09$
- date of onset of symptoms $< 2020/06/01$
- date of death $< 2020/09/01$
- date of death $\geq$ date onset
- date of admission $\geq$ date onset

These filters gave 3 months for *latest possible* onset to resolve (recover or die) and therefore mitigates censoring bias. By filtering date of admission to be greater than onset of symptoms we prevent estimate being biased by nosocomial infections.

In total we obtained 6050 onset-to-death times. We observed a slight trend in onset-to-death with age, but this was uniformly distributed and so it was reasonable to assume a single distribution independent of age. The raw median time onset-death was ~ 11 days, with a mean of ~ 15 days. We fitted a Gamma density function to observed onset-to-death times from daily aggregated CHESS data. Binned data (as opposed to the precise onset-to-death times) were used to facilitate data sharing while preserving anonymity. Little difference was observed when fitting directly on the non-binned data.

Our binned data was of form $(t_i, y_i)_{i=1}^{N}$, where N is the number of binned data observations, $t_i \in \mathbb{Z}$ was an observed number of days between onset and death, and where $y_i \in \mathbb{Z}$ was the number of times that t_i occurred. Thus the total number of onset-to-death times is $T = \sum_{i=1}^{N} y_i = 6050$. Let the probability of dying t days after onset have density function $\pi(t|\theta)$ with parameters θ (e.g. a Gamma distribution with shape and scale or rate parameters). We then define the function, evaluated on day t as $f_t = \int_{x=t-0.5}^{t+0.5} \pi(x,\theta)dx$. Through assuming a Poisson likelihood function $y_t \sim \text{Poisson}(T f_t)$ we can define and fit a

Bayesian hierarchical model.

$$y_t \sim \text{Poisson}(T f_t)$$
$$f_t = \int_{x=t-0.5}^{t+0.5} \text{Gamma}(x, \theta_1, \theta_2) dx$$
$$\theta_1 \sim \mathcal{N}(0,5)$$
$$\theta_2 \sim \mathcal{N}(0,5)$$
$$T = \sum_{i=1}^{N} y_i$$

Note f_t is calculated through cumulative densities, not pointwise, and therefore accounts for the data binning. In our fitting, diffuse $\mathcal{N}(0,5)$ prior distributions were used as hyper parameters of π.

We also tried fitting Frechet, Chi-Square and Log-normal distributions but, using model selection[2], found these to give an inferior fit (Figure S1). The final fitted distribution mean with 95% credible intervals was Gamma$(1.45[1.40-1.50], 10.43[10.01-10.9])$ (Figure S1), with mean 15.17 days and coefficient of variation 0.83.

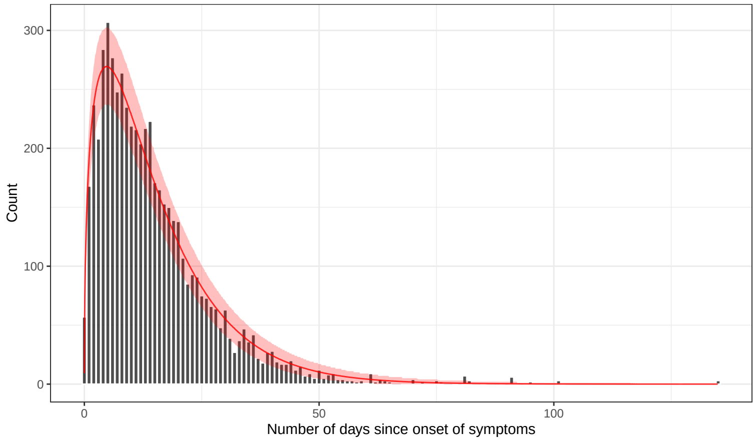

Figure S1. Gamma distributed fitted to onset-to-death times, with 95% credible intervals

By examining empirical survival curves and given the lack of evidence for a lognormal distribution, we found little evidence of long tails. However, because there were observed onset-death times of greater than 100 days, more sensitive density fitting could be investigated. Ultimately, the overwhelming amount of probability mass occurs before this day, and so we believe our fitting sufficient for the results in this paper.

S2 Model Description

We observe $D_{t,m}$ deaths occurring on days $t \in \{1,\ldots,n\}$ and countries $m \in \{1,\ldots,M\}$, where $M = 3$ in this work. Daily deaths are modelled using a positive real-valued function $d_{t,m} = \mathbb{E}[D_{t,m}]$ that represents the expected number of deaths attributed to COVID-19. The daily deaths $D_{t,m}$ are assumed to follow a negative binomial distribution with mean $d_{t,m}$ and variance

$d_{t,m} + \frac{d_{t,m}^2}{\phi}$, where ϕ follows a positive half normal distribution, i.e.

$$D_{t,m} \sim \text{Negative Binomial}\left(d_{t,m}, d_{t,m} + \frac{d_{t,m}^2}{\psi}\right),$$

$$\psi \sim \mathcal{N}^+(0,5).$$

Here, $\mathcal{N}(\mu,\sigma)$ denotes a normal distribution with mean μ and standard deviation σ. We say that X follows a positive half normal distribution $\mathcal{N}^+(0,\sigma)$ if $X \sim |Y|$, where $Y \sim \mathcal{N}(0,\sigma)$. The expected number of deaths d in a given country on a given day is a function of the number of infections (both symptomatic and asymptomatic) c that occurred in previous days.

At the beginning of the epidemic, deaths of individuals infected abroad can bias estimates of the basic reproduction number R_0. To ensure we are modelling deaths from locally acquired infections only, we fit observed deaths from the day after a country has observed a cumulative total of 10 deaths.

To mechanistically link our function for deaths to our latent function for infected cases, we use a previously estimated COVID-19 infection fatality ratio IFR (probability of death given infection) together with a distribution of times from infection to death π. The IFR is based on estimates presented in Verity et al[1]. The country-specific IFRs are assumed to have mean $\text{IFR}_m = 1\%$. To incorporate between-country variability we allow the IFR_m for every country to have some additional, and independent, noise around the mean. This prior distribution therefore puts most of the support (three sigma) of the prior IFR is between $0.7\% - 1.3\%$. Specifically the prior is:

$$\text{IFR}_m^* \sim \text{IFR}_m \cdot N(1, 0.1).$$

Combining estimates from previous studies[1] with new data (see section S1), we assume the distribution of times from infection to death π (infection-to-death) to be the sum of two independent random times: the incubation period (infection to onset of symptoms: "infection-to-onset") distribution and the time between onset of symptoms and death ("onset-to-death"). The infection-to-onset distribution is Gamma distributed with shape 5.8 days and scale 0.94[3]. The onset-to-death distribution is also Gamma distributed with a shape of 1.38 days and scale 10.67 (see section S1). The infection-to-death distribution is therefore given by:

$$\pi \sim \text{Gamma}(5.8, 0.9) + \text{Gamma}(1.45, 10.43).$$

The mean of this distribution is around 20 days. The expected number of deaths $d_{t,m}$, on a given day t, for country, m, is given by the following discrete sum:

$$d_{t,m} = \text{IFR}_m^* \sum_{\tau=0}^{t-1} c_{\tau,m} \pi_{t-\tau},$$

where $c_{\tau,m}$ is the number of new infections on day τ in country m and where π is discretized via $\pi_s = \int_{s-0.5}^{s+0.5} \pi(\tau) d\tau$ for $s = 0, 1, 2, 3, ...$, and where $\pi(\tau)$ is the density of π.

The true number of infected individuals, c, is modelled using a discrete renewal process[4]. To model the number of infections over time, we specify a generation time distribution g with density $g(\tau)$, (the time between when a person becomes infected

and when they subsequently infect another person). The generation time distribution is unknown, but we can approximate it by assuming it is the same as the serial interval distribution (time from symptom onset in one person to the time of symptom onset in the person they infect). We choose both the generation time and the serial interval to be Gamma distributed[5]:

$$g \sim \text{Gamma}(6.5, 0.62).$$

Given the generation time distribution, the number of infections $c_{t,m}$ on a given day t, and country, m, is given by the following discrete convolution function:

$$c_{t,m} = S_{t,m} R_{t,m} \sum_{\tau=0}^{t-1} c_{\tau,m} g_{t-\tau}$$
$$S_{t,m} = 1 - \frac{\sum_{i=1}^{t-1} c_{i,m}}{N_m}$$

where, similar to the probability of death function, the generation time distribution is discretized by $g_s = \int_{s-0.5}^{s+0.5} g(\tau)d\tau$ for $s = 2, 3, ...$, and $g_1 = \int_0^{1.5} g(\tau)d\tau$. The population of country m is denoted by N_m. We include the adjustment factor $S_{t,m} = 1 - \frac{\sum_{i=1}^{t-1} c_{i,m}}{N_m}$ to account for the number of susceptible individuals left in the population: i.e. even in the absence of interventions, herd immunity will reduce the number of daily infected through susceptible depletion. This assumes reinfection over the time horizon of our model is impossible. It is possible to include a correction factor in the serial interval to account for individuals dying before they can infect others However, given the infection-to-death delay distribution this factor is negligible and we have chosen to exclude it. We also note the adjustment factor makes negligible difference to parameter estimation, but excluding it helps greatly improves posterior topology and inference. When generating counterfactual the adjustment factor is always included to account for susceptible depletion.

Infections today depend on the number of infections in previous days, weighted by the discretized generation time distribution. This weighting is then scaled by the country-specific time-varying reproduction number, $R_{t,m}$, which models the average number of secondary infections per infection at a given time. The functional form for the time-varying reproduction number was chosen to be a random process. $R_{t,m}$ is a function defined by:

$$R_{t,m} = e^{\alpha_{0,m} + \mathbb{1}\varepsilon_{w(t),m}} \tag{1}$$
$$R_{t,m} = R_{0,m} e^{\mathbb{1}\varepsilon_{w(t),m}} \tag{2}$$

where the conversion from days to weeks is encoded in $w(t)$. Every 7 days, w is incremented, i.e. we set $w(t) = \lfloor (t - t^{\text{start}})/7 \rfloor + 1$, where t^{start} is set to March 13th. $\varepsilon_{w(t),m}$ is a stochastic process for country m and week $w(t)$. For all $t <$ March 13th, $\varepsilon_{w(t),m}$ is set to zero. This stochastic process is defined to be a discrete random walk process. To specify this process we introduce the parameter $\gamma \sim \mathcal{N}(0, \sigma_0)$ and then model ε as $\varepsilon_{w(t),m} \sim \mathcal{N}(\varepsilon_{w(t)-1,m}, \gamma)$. The exponential form was used to ensure positivity of the reproduction number. The indicator function $\mathbb{1}$ ensures the random walk can only start at a specific date which we set to March 13th. The prior distribution for $\alpha_{0,m}$ was chosen to be

$$\alpha_{0,m} \sim \mathcal{N}(\log(3.5), 0.1)$$
$$R_0 = e^{\alpha_{0,m}}$$

The prior distribution for α_0 was chosen to represent a broad range of plausible reproduction numbers with support from 2 to 5[6]. We assume that seeding of new infections begins on the 30st of January 2020. From this date, we seed our model with

6 sequential days of an equal number of infections: $c_{1,m} = \cdots = c_{6,m} \sim \text{Exponential}(\frac{1}{\tau})$, where $\tau \sim \text{Exponential}(1)$. These seeding infections are inferred in our Bayesian posterior distribution. We estimated parameters jointly for all 11 countries in a single hierarchical model. Fitting was done in the probabilistic programming language Stan[7] using an adaptive Hamiltonian Monte Carlo (HMC) sampler.

Limitations of our model are discussed in detail in[5]. To summarize, we assume:

- a probability distribution with fixed mean and coefficient of variation for infection to symptom onset and from symptom onset to death
- a probability distribution with fixed mean and coefficient of variation for the generation time distribution
- that the generation time and serial interval distributions are the same
- a fixed R_0 for each country with modelled noise
- a fixed average infection fatality ratio mean with additive modelled noise
- average dynamics nationally and across all ages

See section S2 for further details of model assumptions and limitations.

Official data on laboratory-confirmed COVID-19 deaths by date of death were obtained from government websites: for the UK was via the UK government dashboard[8], for Sweden through the Public Health Agency of Sweden (Folkhälsomyndigheten)[9] and for Denmark from the State Serum Institute (Statens Serum Institut)[10]. All of our source code can be found at `https://github.com/ImperialCollegeLondon/covid19model/tree/whatif/whatif`

S3 Counterfactual Specifics and posterior ordering

As introduced in S2 our equation for R_t is

$$R_{t,m} = e^{\alpha_{0,m}+\varepsilon_{w(t),m}} \tag{3}$$

For country m, day t and week w. We can write the actual (i.e. the fitted) R_t for countries x and y

$$R_{t,x} = e^{\alpha_{0,x}} e^{\varepsilon_{w(t),x}} \tag{4}$$

$$R_{t,y} = e^{\alpha_{0,y}} e^{\varepsilon_{w(t),y}} \tag{5}$$

Consider the counterfactual where y is the donor country to recipient country x ($\mathbf{y} \to \mathbf{x}$ in our notation). In our absolute approach, we have

$$R_{t,y\to x} = \begin{cases} e^{\alpha_{0,x}} e^{\varepsilon_{w(t),x}} & \text{if } t < \text{March } 13^{th}, \\ e^{\alpha_{0,y}} e^{\varepsilon_{w(t),y}} & \text{if } t \geq \text{March } 13^{th}. \end{cases}$$

For the relative approach

$$R_{t,y\to x} = \begin{cases} e^{\alpha_{0,x}} e^{\varepsilon_{w(t),x}} & \text{if } t < \text{March } 13^{th}, \\ \frac{e^{\alpha_{0,x}}}{e^{\alpha_{0,y}}} e^{\alpha_{0,y}} e^{\varepsilon_{w(t),y}} & \text{if } t \geq \text{March } 13^{th} \end{cases}$$

and so cancelling terms yields

$$R_{t,y\to x} = \begin{cases} e^{\alpha_{0,x}} e^{\varepsilon_{w(t),x}} & \text{if } t < \text{March } 13^{th}, \\ e^{\alpha_{0,x}} e^{\varepsilon_{w(t),y}} & \text{if } t \geq \text{March } 13^{th} \end{cases}$$

Post prediction ordering

For any given country, posterior samples of the first week of the random walk process are correlated with R_0, but the samples of R_0 for any given country are independent (Figure S2). If naive switching of R_0 is performed then posterior correlations between R_0 and the random walk process are entirely lost (Figure S2) and the resultant uncertainty can be artificially large. There is no rigorous solution to this problem and it arises as a result of independent growth rates in each country. One approach to minimise this problem is to order samples such that high R_0 samples match high random walk samples. This does not change posterior summaries in the fit, but helps maintain some correlation between R_0 and the random walk (Figure S2).

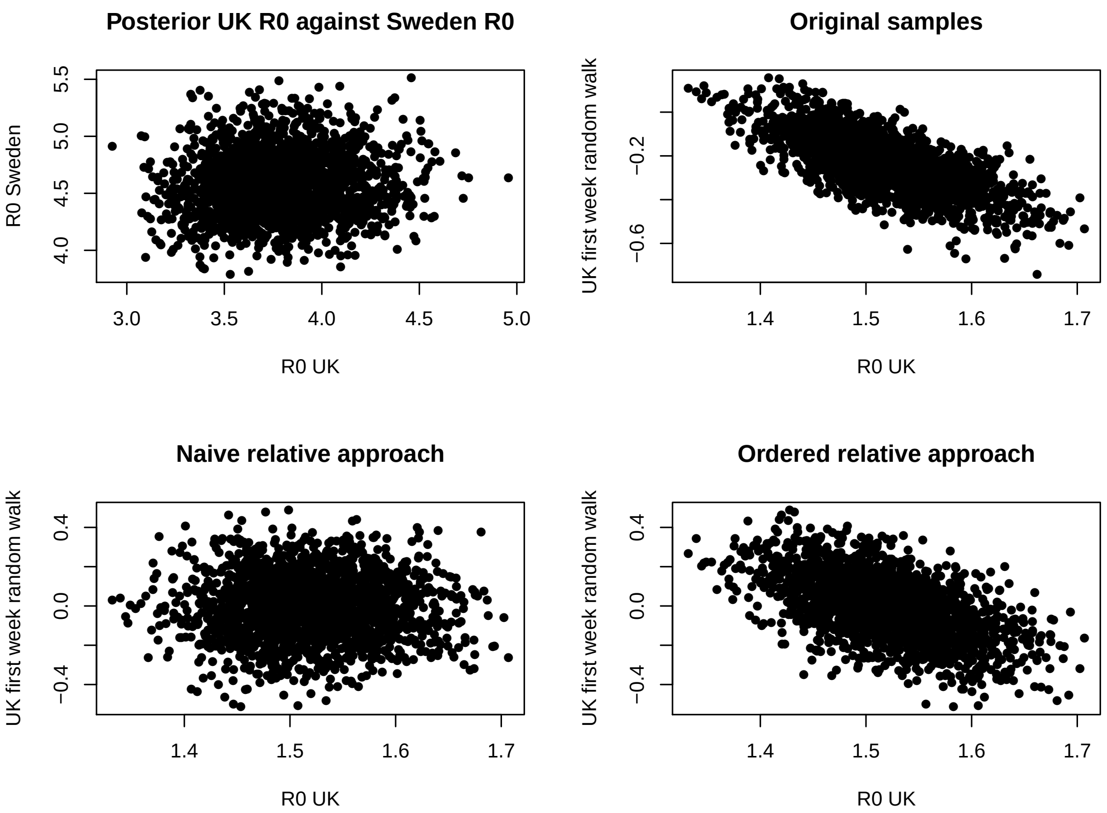

Figure S2. Top left: Joint posterior samples of R_0 for the UK plotted against posterior samples of R_0 from Sweden. Top right: Original correlation structure for the UK for R_0 and the first week of the random walk. Bottom left: Naive scaling of R_0 in the relative structure removes correlation structure for the UK for R_0 and the first week of the random walk. Bottom right: Ordering samples in the scaling of R_0 in the relative structure preserves correlation structure for the UK for R_0 and the first week of the random walk

S4 Original Time-varying Reproduction Plots

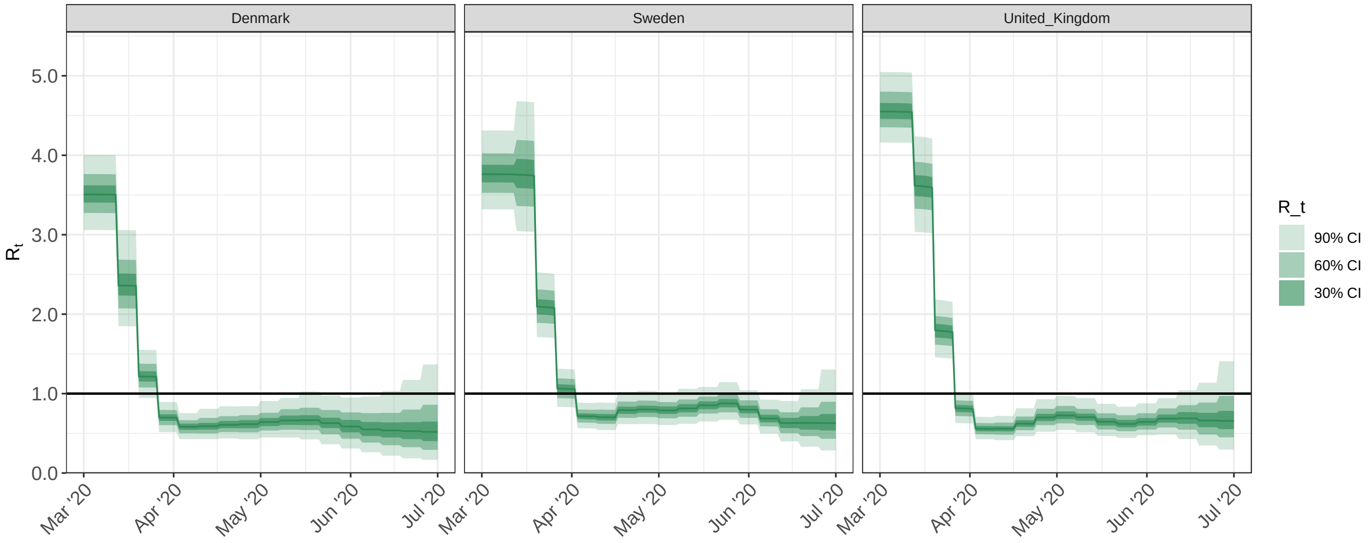

Figure S3. Estimated time-varying reproduction number (R_t) with 95% credible intervals

S5 Total cumulative mortality for counterfactual scenarios

	Recipient Country		
	Denmark	Sweden	United Kingdom
**Donor Country**			
Denmark	606	971 [608-1,591]<br>1,234 [757-2,060]	14,192 [9,683-21,205]]<br>37,866 [24,369-61,039]
Sweden	3,024 [1,892-4,590]<br>2,120 [1,339-3,351]	5,515	66,010 [46,877-91,121]<br>156,038 [117,622-207,557]
United Kingdom	1,604 [1,035-2,338]<br>674 [434-997]	2,626 [1,739-3,891]<br>1,360 [921-1,959]	40,659

Table S1. As per Table 1, but showing absolute mortality until 1st July 2020, and not per-capita deaths.

S6 Sensitivity analysis to infection fatality ratio

In this section we halve the mean IFR from 1% to 0.5%, well below central estimates for the demographics of the countries analysed[1,11,12]. The prior distribution for IFR allows a 3 standard deviation range from 0.35% – 0.65%. Results are very

similar for Sweden and Denmark, but mortality for the UK adopting the Swedish strategy are reduced, because herd immunity has a larger effect in reducing transmission in a high-mortality-but-low-IFR scenario (Table S2). Our substantive conclusions remain unchanged, however.

	**Recipient Country**		
	Denmark	Sweden	United Kingdom
**Donor Country**			
Denmark	105	95 [59-152]<br>122 [72-200]	203 [142-300]<br>516 [352-752]
Sweden	471 [307-703]<br>337 [215-542]	546	832 [622-1132]<br>1,626 [1,288-2,058]
United Kingdom	263 [178-379]<br>113 [75-162]	250 [166-354]<br>133 [87-189]	599

Table S2. As per Table 1. Sensitivity analysis assuming that the infection fatality ration (IFR) is halved. Absolute and relative approach deaths per million estimates shown in red and blue text respectively.

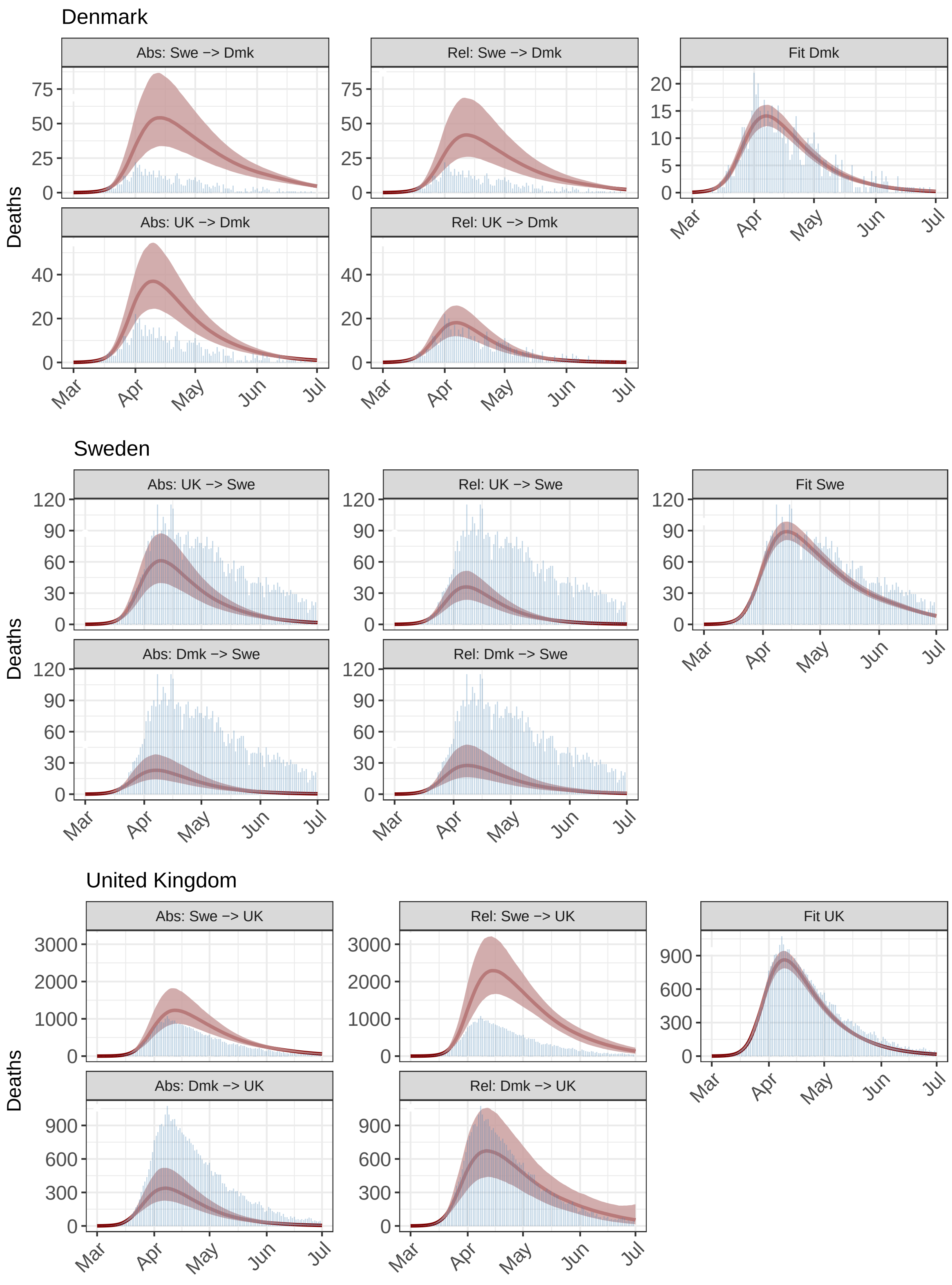

Figure S4. Estimates of daily deaths (red curves with shaded 95% credible intervals). Observed deaths are shown as blue bars. Infection fatality ratio in these estimates has been halved.

S7 Sensitivity analysis to generation time distribution

We investigated the sensitivity of our estimates to the generation time distribution, varying the mean (which takes a value of 6.5 days in our main analysis), to 6, 7 and 8 days. These values were chosen as plausible serial intervals reported from[13]. Tables S3, S4 and S5 show that estimates of deaths per million are highly similar from those reported in our main analysis.

	Recipient Country		
	Denmark	Sweden	United Kingdom
**Donor Country**			
Denmark	105	97 [60-156]<br>124 [77-200]	217 [148-330]<br>519 [340-804]
Sweden	512 [325-789]<br>361 [230-557]	546	988 [724-1391]<br>2042 [1539-2748]
United Kingdom	265 [173-384]<br>120 [78-177]	251 [165-355]<br>141 [94-201]	599

Table S3. As per Table 1. Sensitivity analysis assuming mean generation time of 6 days. Absolute and relative approach deaths per million estimates shown in red and blue text respectively.

	Recipient Country		
	Denmark	Sweden	United Kingdom
**Donor Country**			
Denmark	105	91 [54-149]<br>114 [68-190]	179 [124-269]<br>614 [407-938]
Sweden	564 [346-888]<br>400 [252-645]	546	919 [655-1321]<br>2830 [2148-3734]
United Kingdom	329 [213-473]<br>120 [79-174]	288 [186-422]<br>128 [83-188]	599

Table S4. As per Table 1. Sensitivity analysis assuming mean generation time of 7 days. Absolute and relative approach deaths per million estimates shown in red and blue text respectively.

	Recipient Country		
	Denmark	Sweden	United Kingdom
**Donor Country**			
Denmark	105	99 [60-164]<br>123 [72-206]	234 [160-359]<br>401 [257-654]
Sweden	497 [313-780]<br>362 [222-581]	546	1009 [743-1401]<br>1510 [1044-2123]
United Kingdom	244 [158-358]<br>139 [86-215]	233 [155-340]<br>166 [107-249]	599

Table S5. As per Table 1. Sensitivity analysis assuming mean generation time of 8 days. Absolute and relative approach deaths per million estimates shown in red and blue text respectively.

S8 Sensitivity analysis to onset-to-death distribution

We investigated our estimates' sensitivity to our fitted onset-to-death distribution, varying its mean from a value of 15.17 days, to 11, 13, 17 and 19 days (Tables S6, S7, S8, and S9 respectively). In all scenarios, deaths per million are similar to those reported in the main text.

	Recipient Country		
	Denmark	Sweden	United Kingdom
**Donor Country**			
Denmark	105	97 [59-163]<br>129 [79-217]	208 [139-314]<br>699 [448-1113]
Sweden	514 [317-799]<br>345 [210-542]	546	956 [692-1313]<br>2465 [1864-3192]
United Kingdom	278 [179-409]<br>102 [64-154]	261 [177-380]<br>122 [81-176]	599

Table S6. As per Table 1. Sensitivity analysis assuming mean of 11 days in onset-to-death distribution. Absolute and relative approach deaths per million estimates shown in red and blue text respectively.

	Recipient Country		
	Denmark	Sweden	United Kingdom
**Donor Country**			
Denmark	105	95 [58-154]<br>124 [76-209]	207 [141-306]<br>622 [401-985]
Sweden	524 [331-807]<br>362 [216-560]	546	976 [698-1322]<br>2357 [1765-3134]
United Kingdom	279 [182-403]<br>109 [70-165]	256 [175-379]<br>128 [88-187]	599

Table S7. As per Table 1. Sensitivity analysis assuming mean of 13 days in onset-to-death distribution. Absolute and relative approach deaths per million estimates shown in red and blue text respectively.

	Recipient Country		
	Denmark	Sweden	United Kingdom
**Donor Country**			
Denmark	105	95 [58-153]<br>116 [72-196]	206 [143-312]<br>497 [327-772]
Sweden	530 [343-832]<br>389 [237-607]	546	974 [711-1363]<br>2224 [1690-2982]
United Kingdom	279 [182-412]<br>125 [83-182]	257 [172-377]<br>140 [93-200]	599

Table S8. As per Table 1. Sensitivity analysis assuming mean of 17 days in onset-to-death distribution. Absolute and relative approach deaths per million estimates shown in red and blue text respectively.

	Recipient Country		
	Denmark	Sweden	United Kingdom
**Donor Country**			
Denmark	105	93 [59-154]<br>114 [69-191]	206 [140-315]<br>455 [295-706]
Sweden	537 [332-842]<br>391 [236-619]	546	985 [711-1396]<br>2176 [1616-2938]
United Kingdom	282 [182-421]<br>131 [85-194]	259 [169-373]<br>144 [97-214]	599

Table S9. As per Table 1. Sensitivity analysis assuming mean of 19 days in onset-to-death distribution. Absolute and relative approach deaths per million estimates shown in red and blue text respectively.

S9 MCMC specifics and Diagnostic Summary

Adaptive Hamiltonian Monte Carlo[7] was used for posterior inference with 1000 iterations (500 warm up 500 sampling) over 4 chains, with target acceptance rates of 0.95 and tree depth of 15.

Posterior predictive checks for our fits all show a well calibrated posterior. Trace files for all chains, along with R-hat statistics (see Figure S7) indicated convergence, well mixed chains, and no pathological behaviour. We summarise here two metrics of this performance relevant to Hamiltonian Monte Carlo[14]. Figure S5 shows the energy plot. identifying the presence of overly heavy tails that are challenging for sampling or that suggest high autocorrelation. The similarity of the histograms suggests no pathological behaviour. Figure S6 shows that no divergent transitions were observed, suggesting good exploration of the target posterior distribution. Low acceptance rates and concentrated log posterior scores would indicate that parts the posterior were not explored.

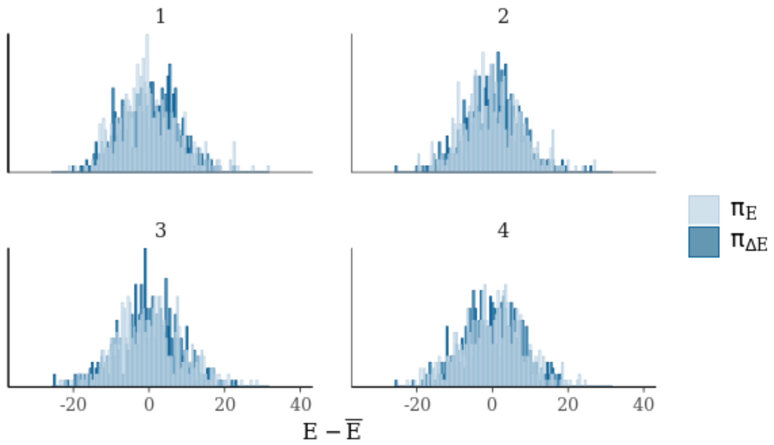

Figure S5. Energy plot. The momentum resampling in a Hamiltonian Markov transition induces a change of energies and a walk across level sets. The figure shows energy distribution induced by momentum resampling is similar to the marginal energy distribution suggesting rapid exploration of the posterior and minimal autocorrelation.

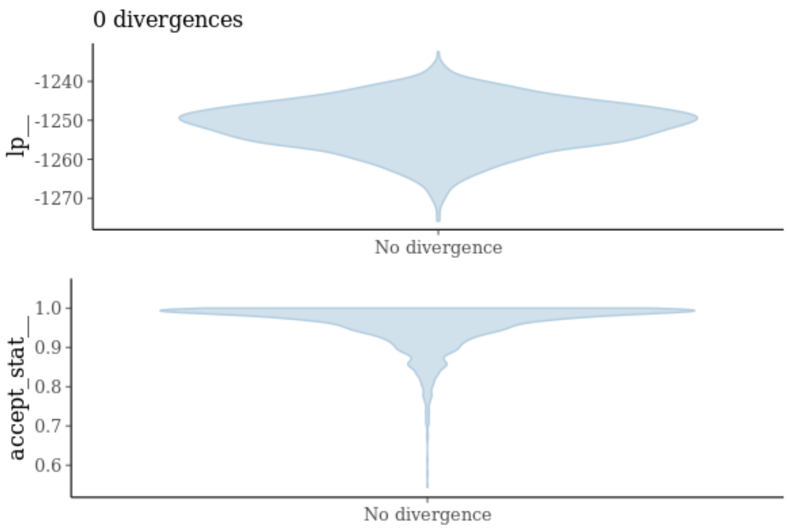

Figure S6. Divergence plot. Divergences often indicate that some part of the posterior distribution is not being explored. These plots show our model has no divergent transitions and a high acceptance rate.

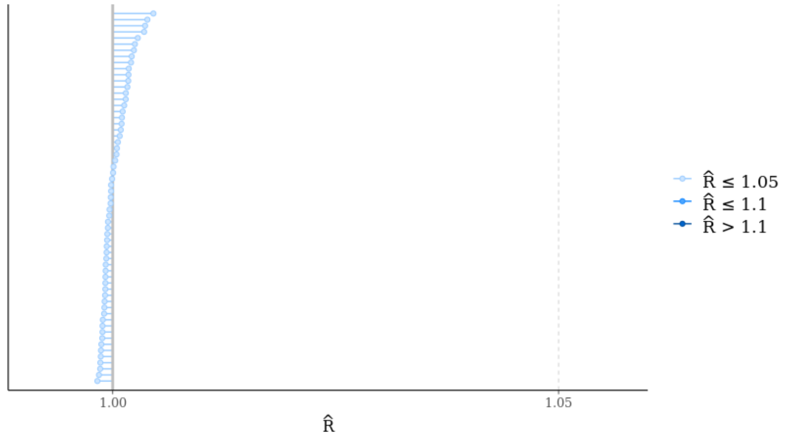

Figure S7. The degree of convergence of a random Markov chain can be estimated using the Gelman-Rubin convergence statistic. Values close to one indicate convergence to the underlying distribution. Values greater than 1.1 indicate inadequate convergence.

References

1. Verity, R. *et al.* Estimates of the severity of {covid}-19 disease. *Lancet Infect. Dis.* **in press** (2020).
2. Vehtari, A., Gelman, A. & Gabry, J. Practical bayesian model evaluation using leave-one-out cross-validation and WAIC. *Stat. Comput.* **27**, 1413–1432 (2017).
3. Lauer, S. A. *et al.* The incubation period of coronavirus disease 2019 (COVID-19) from publicly reported confirmed cases: Estimation and application (2020).
4. Mishra, S. *et al.* On the derivation of the renewal equation from an age-dependent branching process: an epidemic modelling perspective. *arXiv* (2020). 2006.16487.
5. Flaxman, S. *et al.* Estimating the effects of non-pharmaceutical interventions on COVID-19 in europe. *Nature* (2020).
6. Liu, Y., Gayle, A. A., Wilder-Smith, A. & Rocklöv, J. The reproductive number of COVID-19 is higher compared to SARS coronavirus. *J. Travel. Med.* **27** (2020).
7. Carpenter, B. *et al.* Stan: A probabilistic programming language. *J. Stat. Softw.* **76** (2017).
8. Coronavirus (COVID-19) in the UK: UK Summary (2020). [Online; accessed 25. Sep. 2020].
9. Covid-19, F. (2020). [Online; accessed 23. Sep. 2020].
10. COVID-19, S. S. I. U. M. (2020). [Online; accessed 23. Sep. 2020].
11. Grewelle, R. E. & De Leo, G. A. Estimating the Global Infection Fatality Rate of COVID-19. *medRxiv* 2020.05.11.20098780 (2020). 2020.05.11.20098780.
12. Russell, T. W. *et al.* Estimating the infection and case fatality ratio for coronavirus disease (COVID-19) using age-adjusted data from the outbreak on the diamond princess cruise ship, february 2020. *Euro Surveill.* **25** (2020).

13. Bi, Q. *et al.* Epidemiology and transmission of COVID-19 in 391 cases and 1286 of their close contacts in shenzhen, china: a retrospective cohort study. *Lancet Infect. Dis.* **20**, 911–919 (2020).

14. Betancourt, M., Byrne, S., Livingstone, S. & Girolami, M. The geometric foundations of Hamiltonian Monte Carlo. *Bernoulli* **23**, 2257–2298, DOI: 10.3150/16-BEJ810 (2017).
